# Linear epitopes of bony fish β-parvalbumins

**DOI:** 10.3389/fimmu.2024.1293793

**Published:** 2024-03-05

**Authors:** Eric Franciskovic, Linnea Thörnqvist, Lennart Greiff, Maria Gasset, Mats Ohlin

**Affiliations:** ^1^ Department of Immunotechnology, Lund University, Lund, Sweden; ^2^ Department of Otorhinolaryngology, Head & Neck Surgery, Skåne University Hospital, Lund, Sweden; ^3^ Department of Clinical Sciences, Lund University, Lund, Sweden; ^4^ Institute of Physical-Chemistry Blas Cabrera, Spanish National Research Council, Madrid, Spain; ^5^ SciLifeLab, Lund University, Lund, Sweden

**Keywords:** allergen, epitope, fish, microarray, parvalbumin, peptide microarray

## Abstract

**Introduction:**

Fish β-parvalbumins are common targets of allergy-causing immunity. The nature of antibody responses to such allergens determines the biological outcome following exposure to fish. Specific epitopes on these allergens recognised by antibodies are incompletely characterised.

**Methods:**

High-content peptide microarrays offer a solution to the identification of linear epitopes recognised by antibodies. We characterized IgG and IgG4 recognition of linear epitopes of fish β-parvalbumins defined in the WHO/IUIS allergen database as such responses hold the potential to counter an allergic reaction to these allergens. Peripheral blood samples, collected over three years, of 15 atopic but not fish-allergic subjects were investigated using a microarray platform that carried every possible 16-mer peptide of known isoforms and isoallergens of these and other allergens.

**Results:**

Interindividual differences in epitope recognition patterns were observed. In contrast, reactivity patterns in a given individual were by comparison more stable during the 3 years-course of the study. Nevertheless, evidence of the induction of novel specificities over time was identified across multiple regions of the allergens. Particularly reactive epitopes were identified in the D helix of Cyp c 1 and in the C-terminus of Gad c 1 and Gad m 1.02. Residues important for the recognition of certain linear epitopes were identified. Patterns of differential recognition of isoallergens were observed in some subjects.

**Conclusions:**

Altogether, comprehensive analysis of antibody recognition of linear epitopes of multiple allergens enables characterisation of the nature of the antibody responses targeting this important set of food allergens.

## 1 Introduction

**Figure 1:**
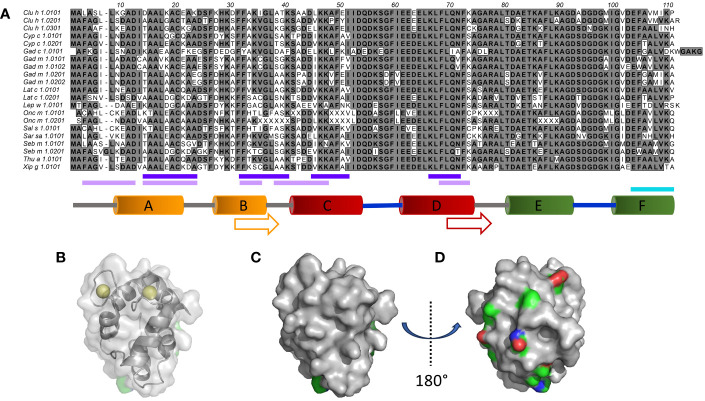
Sequence and structural diversity of fish β-parvalbumins. **(A)** Alignment of 21 fish β-parvalbumin sequences. Residues are defined using the standard IUPAC one-letter code. The predicted B-cell epitopes [http://tools.iedb.org/bcell/ (6);] and IgE reported epitopes of Gad m 1.0202 (7) and Gad m 1 (8) are underlined in light and dark purple, and cyan, respectively. The regions making up the six α-helices in the crystal structure of Gad m 1 (PDB: 2MBX) are depicted by orange (A, B), red (C, D), and green (E, F) cylinders and the calcium-binding loops as thick blue lines. Sequence cores of the regions displaying amyloid folds as synthetic peptides are shown by arrows (7). **(B–D).** The structure of Gad m 1 (PDB: 2MBX) illustrates two faces of parvalbumins, one towards the less diverse surface close to the Ca^2+^ ion coordination sites (**B** (also illustrating the coordinated ions), and **C**), and another towards the face made up of the loops and the A and B helices that make up the N-terminal-most EF-hand motif of parvalbumins **(D)**. Subfigure **D** has been rotated 180° around the y-axis relative to subfigures **(B, C)**. Side chain carbons (green), nitrogens (blue), and oxygens (red) of residues for which the frequency of the most prominent amino acid is <50% are highlighted.

Fish is considered one of the major food allergen sources (1), alongside milk, egg, wheat, peanuts, tree nuts, shellfish, sesame, and soybeans, and its presence in foods must commonly, depending on local legislation (2), be declared. β-Parvalbumins (3) represent the major type of fish allergen (4). They are low molecular weight Ca^2+^-binding proteins that carry three EF-hand units (AB, CD, and EF), two of which typically bind calcium (5) (**Figure 1**). Each such EF-hand consists of two α-helices linked together via a loop.

Fish allergies are initiated by the immune response involving the production of allergen-specific immunoglobulin E (IgE), an antibody isotype present at low concentration in serum and attached to IgE receptors on basophils and mast cells. Properties such as affinity, specificity, and clonal complexity of humoral immune responses to allergens are key features that define the biological outcome of an antibody-allergen interaction (9–11). Allergen-specific antibody responses of the IgE isotype, but also those of other isotypes, in particular IgG and its IgG4 subclass, and IgA, which are commonly associated with protection against allergy (12, 13), will have an impact on the biomedical outcome of the antibody-allergen interaction. Commonly employed clinical tests assess binding to complex allergen extracts, the content of which may be variable and inconsistent (14), or in some cases recombinant allergens. Allergen extracts may lack multiple important allergens (14), and diagnostic assays based on recombinant allergens will not cover all isoallergens and isoforms of allergens. Food processing prior to consumption and environmental changes to the allergen introduced by the oral route of exposure may alter the immunochemical nature of food allergens (15) in ways not captured by common diagnostic assays. Furthermore, diagnostic tests do not address the complexity of the environmental changes of the route of exposure or the response at epitope resolution. Indeed, epitope localization has been implied as a factor that influences the biological outcome of antibody-allergen interaction (16). Altogether, several aspects complicate the interpretation of common diagnostic assays, and there is a need for multiple assay types to describe immune recognition of allergens in fine detail.

Deconvolution of epitope specificities may allow for an enhanced personalized interpretation of the response to an allergen, a feat not achieved by most assay systems in common use. However, a few epitopes of fish parvalbumins have been identified (15). Epitope mapping can be performed by a multitude of techniques like X-ray crystallography, NMR, hydrogen-deuterium exchange mass spectrometry (HDX-MS), and alanine scanning mutagenesis of individual allergens. However, such methodologies are typically not readily applicable in clinical settings and/or cannot be deployed with sufficient throughput as we need to assess reactivities to the large numbers of isoallergens and allergen isoforms (definition by Pomés et al. (17)) that may be of relevance to allergy. *In silico* prediction of conformational epitopes is investigated as an alternative strategy to define epitopes of antibodies, but benchmarking of the performance of such tools has not been encouraging (18).

Allergen peptide arrays are employed to define the complexity and nature of immune responses relevant to allergies, including that of a few fish parvalbumins (8, 19). Although peptide mapping technology cannot efficiently address conformational epitopes recognised by antibodies, it can directly visualise the nature of the subsection of humoral immunity that targets conformation-independent epitopes. Importantly, peptide arrays can be scaled to cover a large diversity of allergens in a high-throughput format. Such technology is used by us in efforts to identify epitopes of several other allergens (20, 21). As an alternative high-throughput technology, a recent study employed immunoprecipitation of allergen peptides displayed on T7 phage and subsequent sequencing-based deconvolution of genes encoding peptides bound by human antibodies, to enable identification of epitopes recognized by human antibodies (22).

A microarray platform that covers essentially every linear 16-mer peptide of all isoallergens and allergen isoforms annotated in 2014 by the WHO/IUIS Allergen Nomenclature Sub-Committee (https://www.allergen.org) was recently developed (20). The platform carries peptides of 21 β-parvalbumin sequences derived from twelve bony fish species. This system has been used in the present study to analyse the linear epitope profile of humoral immunity towards fish parvalbumins in a cohort composed of 15 atopic individuals who are not allergic to fish. Their β-parvalbumin-specific antibody levels were studied over 1-3 years, as a first step towards the definition of the complexity of linear epitopes of these important allergens as recognised by IgG with the capacity to protect against allergic disease.

## 2 Materials and methods

### 2.1 Fish β-parvalbumin linear epitopes detection by the peptide microarray

Sequences of all 21 fish β-parvalbumins (**Table 1**), present in the WHO/IUIS Allergen Nomenclature Sub-Committee’s Allergen Nomenclature Database (http://allergen.org/) in 2014, had been included in a peptide microarray covering 731 allergens, and 1105 isoallergens and isoforms thereof, as outlined by Mikus et al. (20). The peptide sequences of Onc m 1 isoallergens were incomplete as they were incomplete in the database. The array also carries peptides of the β-parvalbumin of *Rana esculenta* Ran e 2 (edible frog). The microarray covered every possible linear 16-mer peptide of these parvalbumins. Serum samples of 15 subjects (age 22-47 at the start of the study; women/men: 7/8) allergic to aeroallergens had been collected at Lund University Hospital (Lund, Sweden) (23, 24). The study was performed according to the Declaration of Helsinki, approved by the regional ethical board at Lund University, and written informed consent was obtained from study participants. These samples were analysed for their ability to recognise linear allergen epitopes (20, 21). Eight of these subjects (Donors 1-8) had, in a study unrelated to the present research question, undergone specific immunotherapy to treat aeroallergen allergy. Consecutive samples were collected before initiation of treatment (sample 1), during immunotherapy up-dosing (these samples were not collected from individuals that did not undergo immunotherapy) (sample 2), and after one (sample 3) and three (sample 4) years. The sample at year 3 was not collected from subjects 1, 9, 10, 14, and 15. No allergy to fish had been recorded. Binding of IgE, IgG, and IgG4 to all peptides on the microarray has been determined as described by Mikus et al. (20). All peptide sets but those that represent Gad c 1.0101 and isoallergens of Onc m 1 include a peptide carrying the N-terminal methionine.

**Table 1:** Fish β-parvalbumin allergens represented on the peptide microarray.

Fish specie		Allergen
Atlantic herring	*Clupea harengus*	Clu h 1.0101, Clu h 1.0201, Clu h 1.0301
Common carp	*Cyprinus carpio*	Cyp c 1.0101, Cyp c 1.0201
Baltic cod	*Gadus callarias*	Gad c 1.0101
Atlantic cod	*Gadus morhua*	Gad m 1.0101, Gad m 1.0102, Gad m 1.0201, Gad m 1.0202
Barramundi	*Lates calcarifer*	Lat c 1.0101, Lat c 1.0201
Megrim	*Lepidorhombus whiffiagonis*	Lep w 1.0101
Rainbow trout	*Oncorhynchus mykiss*	Onc m 1.0101*, Onc m 1.0201*
Atlantic salmon	*Salmo salar*	Sal s 1.0101
Pacific pilchard	*Sardinops sagax*	Sar sa 1.0101
Ocean perch	*Sebastes marinus*	Seb m 1.0101, Seb m 1.0201
Yellowfin tuna	*Thunnus albacares*	Thu a 1.0101
Swordfish	*Xiphias gladius*	Xip g 1.0101

* These allergens are incomplete as some of their residues are not defined in the WHO/IUIS Allergen Nomenclature Sub-Committee’s Allergen Nomenclature Database. Consequently, they are not included in the present analysis.

### 2.2 Epitope prediction

Epitope prediction was performed using the Kolaskar & Tongaonkar Antigenicity methodology (6) as featured by the Antibody Epitope Prediction tool (http://tools.iedb.org/bcell/) of the Immune Epitope Database Analysis Resource (25).

### 2.3 Protein structures

Protein structures of Gad m 1 (PDB: 2MBX) and Cyp c 1 (PDB: 1B8R) were downloaded from the Protein Data Bank (https://www.rcsb.org/). Protein structures were visualized using PyMol (The PyMOL Molecular Graphics System, Version 2.2.3 Schrödinger, LLC).

### 2.4 Principal component analysis

Principal component analysis (PCA) calculations were performed in OmicLoupe v. 1.1.7 (26) using log base 2-converted fluorescence intensity values representing IgG binding to peptides. Reactivity values of peptides starting at position 9-95 (as defined in **Figure 1**) were used to compare reactivity profiles to the 19 β-parvalbumin allergens. Reactivity values of the 1412 unique peptides that represent these β-parvalbumin allergens were used to assess the relationship of reactivity profiles of all blood samples investigated in this study.

### 2.5 Cluster heatmap

Heatmaps and clustering were drawn with R function “pheatmap” from the pheatmap package (version 1.0.12) (https://CRAN.R-project.org/package=pheatmap). Fluorescence intensity values for IgG binding to peptides starting at position 1 (as defined in **Figure 1**) of the allergens were first converted to log base 2 values and then organised into a dataframe suited to the tool. Clustering was enabled only per sample.

## 3 Results

### 3.1 Fish parvalbumin linear epitopes – general overview of study design

**Figure 2:**
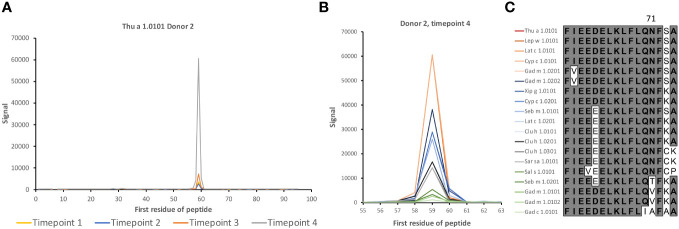
IgG4 antibody reactivity in donor 2 to an epitope on β-parvalbumin. **(A)** Increased levels of IgG4 reactivity were observed during the 3-year study period to peptides of the C-terminus of the CD loop and the D-helix of β-parvalbumins (core epitope starting at residue 59 (as defined in **Figure 1**), represented in Thu a 1.0101 by sequence FIEEDELKLFLQNFSA. **(B, C)**. Intensity of reactivity to the core peptide varied between peptides of different allergens (see also **Supplementary Figure 1**). Species that carry identical peptides at position 59 will show only once in **(B)** The binding pattern suggests that N71, a surface-exposed residue, is important for epitope recognition in this subject. The numbers of the first residue of peptides are defined in **Figure 1**.

High-content 16-mer peptide microarrays were used to map linear epitope profiles found in the serum of 15 human subjects with a history of allergy to aeroallergens, but not specifically against fish. The epitope profiles will likely represent the focus of humoral immunity to fish allergens in non-allergic subjects. The microarray fully included peptides representing β-parvalbumin of 11 fish species and 19 isoallergens/isoforms thereof (**Table 1**). The most diverse residues were located towards the N-terminal part of β-parvalbumin, primarily up to the beginning of the C-helix (**Figure 1**). As a result, the sequence diversity of β-parvalbumins focused on one face of these proteins (**Figures 1C, D**). Very limited, if any, antibody reactivity of IgE class was as expected seen against fish beta-parvalbumin for the 15 studied, not fish allergic subjects. In contrast, extensive immunoreactivity was typically seen for IgG antibodies. Only a small portion thereof appeared to represent IgG4 reactivity; nevertheless, strong IgG4 reactivity was seen in a few samples at regions overlapping the core of a previously reported Gad m 1.0202 IgE epitope (7) (**Figure 2**; **Supplementary Figure 1**).

**Figure 3:**
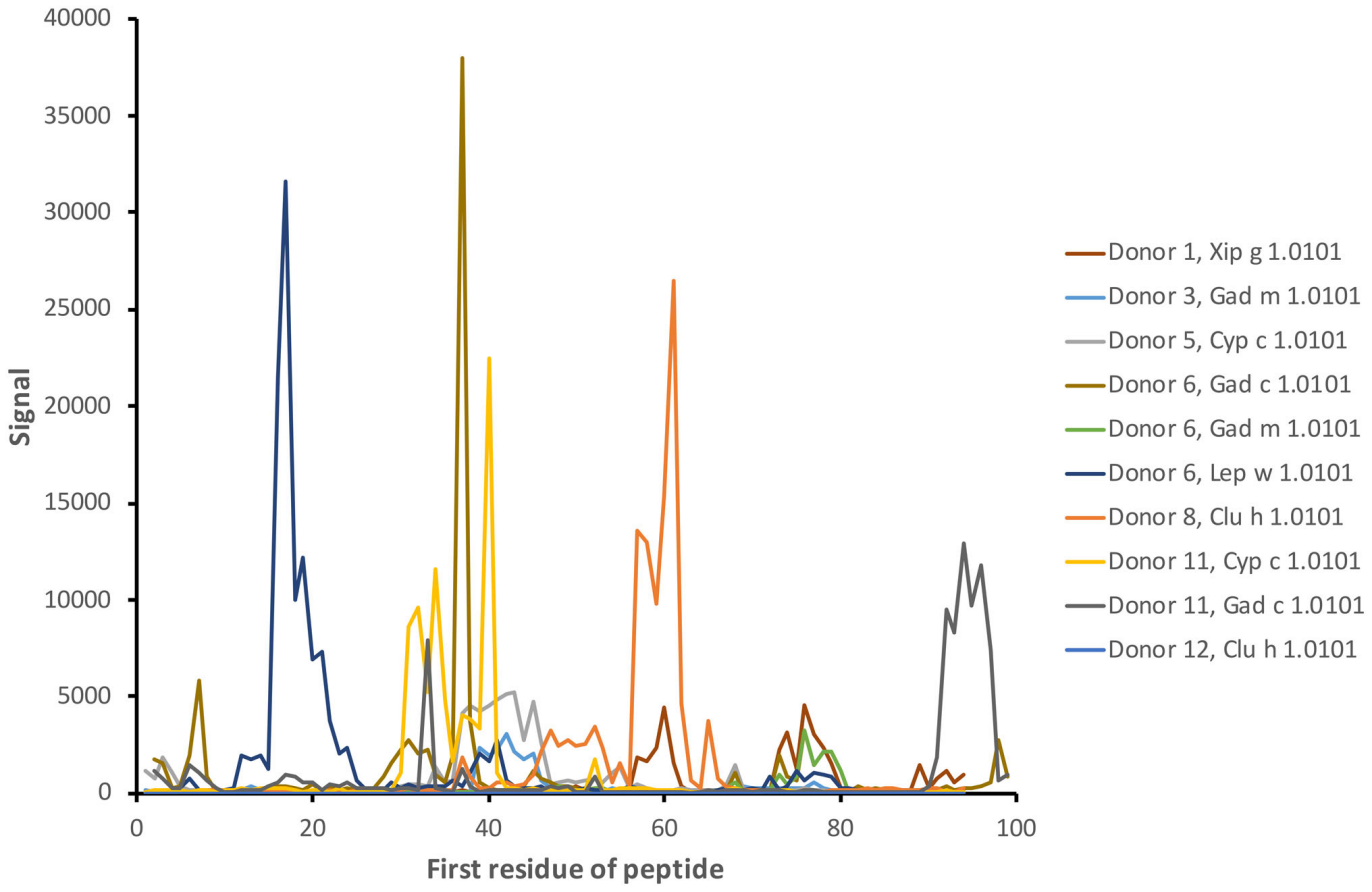
Examples of antibody reactivity (IgG) targeting diverse linear epitopes along the sequence of fish β-parvalbumins. The examples represent the reactivity of samples collected at the start of the study (time point 1). The numbers of the first residue of peptides are defined in **Figure 1**.

**Figure 4:**
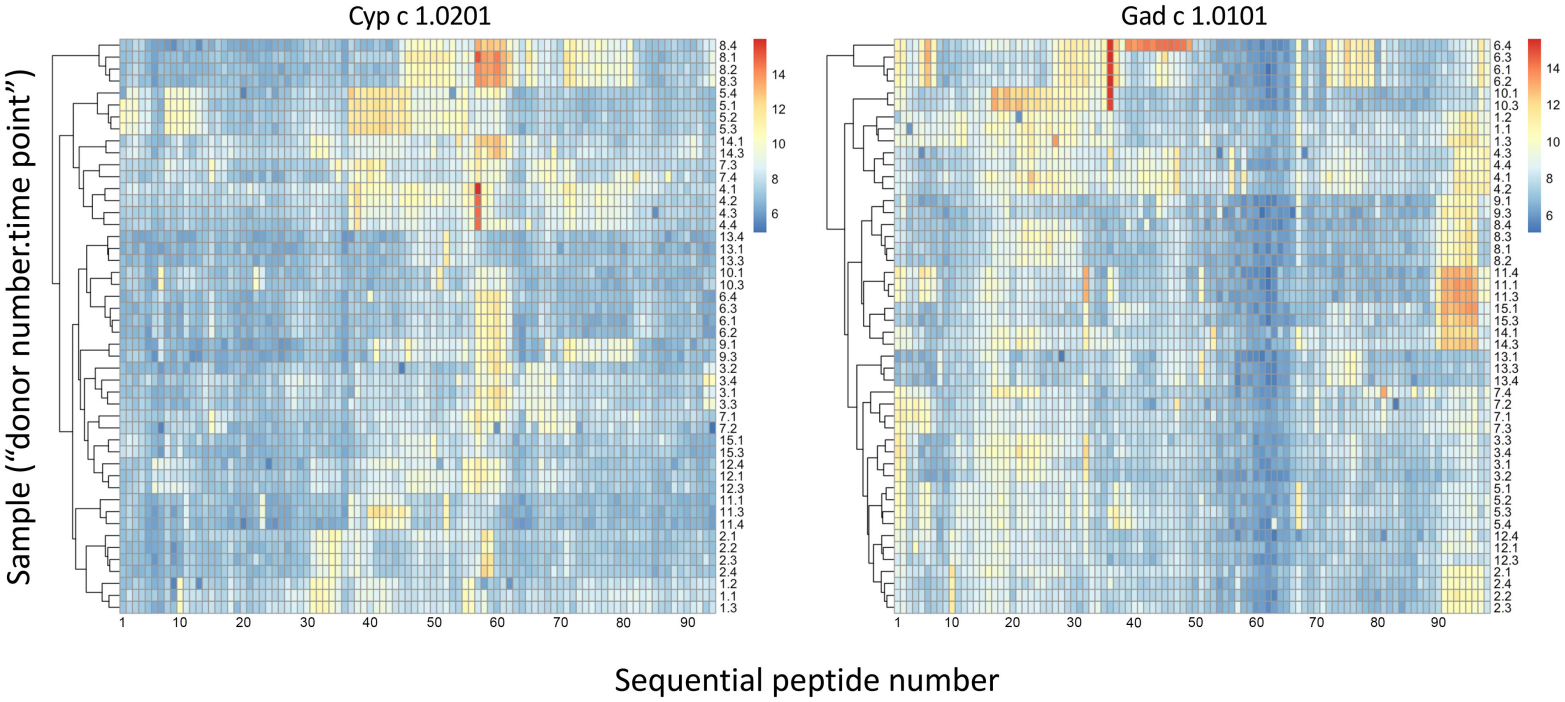
Heat maps of IgG binding to peptides of Cyp c 1.0201 and Gad c 1.0101, illustrating clustering and similarity of reactivity profiles of consecutive samples of each individual during a 3-year study period (naming: “donor number 1-15”; “sample time point 1-4”). Major epitopes around peptide 58 and close to the C-terminus, respectively, of Cyp c 1.0201 and Gad c 1.0101, respectively, are evident. Reactivity profiles of other allergens are shown in **Supplementary Figure 2**. Based on the representation of these allergens in the IUIS allergen database, the first peptide of Cyp c 1.0201 and Gad c 1.0101 starts with the N-terminal methionine and the subsequent alanine, respectively. Numbers represent the linear order of peptides of each allergen.

**Figure 5:**
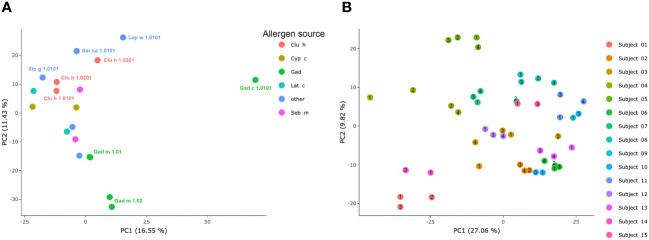
PCA of IgG-responses to fish parvalbumins. **(A)** Similarity of immunoreactivity profiles targeting individual allergens across the set of allergens. **(B)** Similarity of samples derived from each individual during the study period. Each sample’s time point (1–4) is indicated by a number within or immediately adjacent to each dot.

Linear epitopes recognized by the subject’s IgG antibodies were distributed to diverse but discrete locations along the β-parvalbumin sequences (**Figure 3**). In many cases, epitopes extended across multiple adjacent peptides, but exceptions occurred requiring the full 16-mer sequence of a peptide to fully represent an epitope (such as an epitope of Gad c 1.0101 recognised by IgG of donors 6 and 10) (**Figure 4**; **Supplementary Figure 2**). The fact that such epitopes typically were seen in multiple consecutive samples (**Figure 4**; **Supplementary Figure 2**), and with multiple similar peptides from different allergens/isoforms/isoallergens, strongly suggests that the observed reactivities indeed represented specific antibody binding, and not a local technical artifact on the peptide microarray surface. In the studied cohort it was observed that immunoreactivity profiles targeting each allergen were stable for a given subject over the 3-year study period (**Figure 4**; **Supplementary Figure 2**). PCA of IgG reactivity against the complete set of β-parvalbumins likewise showed that samples collected from the same individual at different time points grouped (**Figure 5B**).

### 3.2 Immunological similarity of fish β-parvalbumins

**Figure 6:**
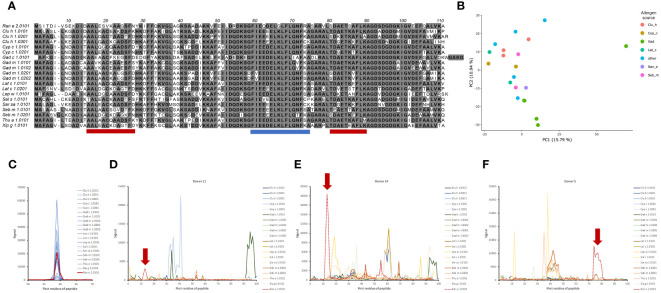
Reactivity of serum IgG to peptides of frog β-parvalbumin allergen Ran e 2.0101. **(A)** Sequence of frog allergen Ran e 2.0101 in comparison to fish β-parvalbumins. Three linear epitopes, further described in **(C–F)**, of Ran e 2.0101 are indicated by blue **(C)** and red **(D–F)** bars. **(B)** PCA plot demonstrating the similarity of antibody recognition of Ran e 2 to that of multiple other allergens, including *e.g.* Gad m 1.01 C. Specific IgG4 of subject 2 (see **Figure 2** for full details of the fish allergen reactivity) that cross-react to multiple fish β-parvalbumins (blue) also cross-react to the corresponding epitope (red) of Ran e 2.0101. **(D–F)**. Peptide reactivity profiles of serum IgG of three subjects (representative samples collected at the start of the study are shown) to β-parvalbumins including Ran e 2.0101 are shown. Epitopes (outlined in red in **(A)**) primarily or only recognized in peptides of Ran e 2.0101 are highlighted by red arrows.

To assess the overall similarity of the antibody recognition patterns of the diverse β-parvalbumins analysed in the present study, we assessed, using PCA, the similarity of antibody responses across all subjects of the study. While most allergens grouped, Gad c 1 and to some extent Gad m 1.02 (but not Gad m 1.01) had epitope profiles separated from those of other allergens and each other (**Figure 5A**). The distinct binding profile of Gad c 1 was evident in most subjects investigated in this study (**Supplementary Figure 3**) Antibody reactivity to β-parvalbumin (Ran e 2.0101) of the phylogenetically distant edible frog largely clustered with that of most fish β-parvalbumin (**Figure 6**). For instance, the IgG4-reactivity to a cross-reactive epitope also recognized the corresponding peptide of Ran e 2.0101 (**Figure 6C**). In a few cases additional epitopes not identified as major epitopes of fish β-parvalbumins were found in this frog antigen (**Figures 6D-F**), suggesting that other β-parvalbumins than those of fish may be inducing immunoreactivity to β-parvalbumins in human subjects.

### 3.3 Immunodominant epitopes in fish β-parvalbumins

**Figure 7:**
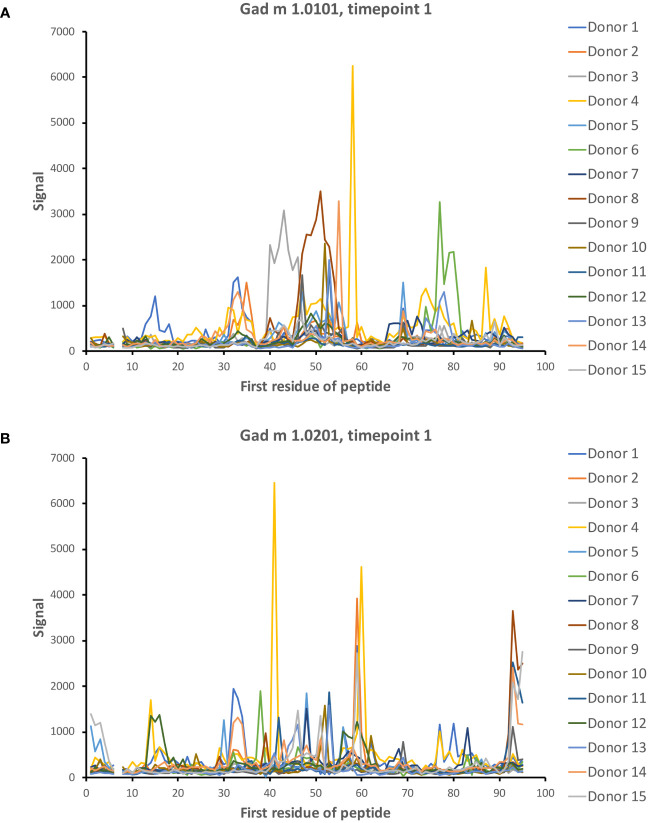
IgG seroreactivity to peptides of isoallergens of Gad m 1. Reactivity is observed to several peptides. Peptides of the C-terminal part of Gad m 1.0201 **(B)** but not Gad m 1.0101 **(A)** are recognized by 5/15 patient sera. The numbers of the first residue of peptides are defined in **Figure 1**.

Dominant epitopes were identified in several allergens and preferentially identified epitopes that differed even between similar antigens. In Gad c 1.0101, epitopes commonly recognized by IgG were located at the C-terminal part of the B-helix and the BC junction (8/15 donors) and at the F-helix (8/15 donors) (**Figure 4**; **Supplementary Figure 4**). The C-terminal part of Gad m 1.02 (but not Gad m 1.01) was also recognized by antibodies of several sera (**Figure 7**; **Supplementary Figure 2**). Cyp c 1.0201 tended to be recognized by antibodies specific for an epitope in the D-helix (in at least 8/15 donors) (**Figure 4**). In contrast, this region was particularly cold in Gad c 1.0101 in terms of antibody recognition (**Figure 4**) possibly relating to its unique isoleucine-alanine motif vs. the common glutamine-asparagine sequence motif seen in most investigated β-parvalbumin at residues 70-71 (**Figure 1A**). In conclusion, different β-parvalbumins may be primarily targeted by antibodies against different epitopes.

### 3.4 Induction of novel reactivities during the study period

**Figure 8:**
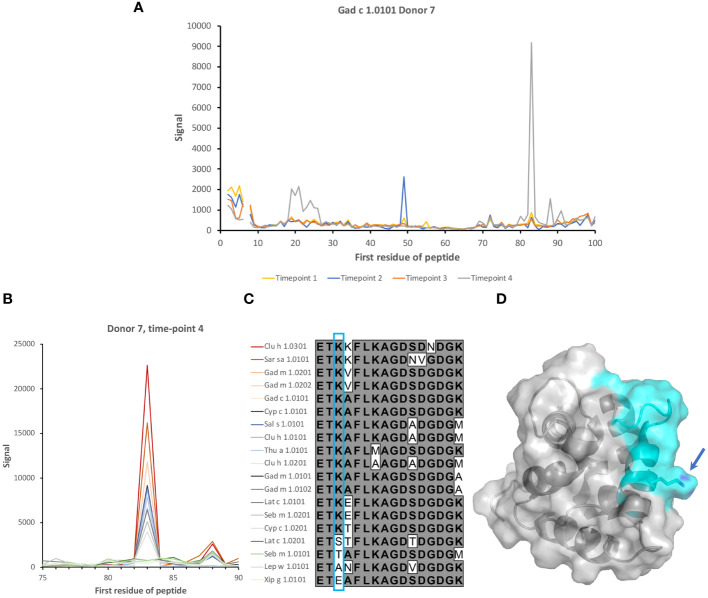
Induction of recognition of two epitopes of β-parvalbumins during the study period. **(A)** Reactivity profile of samples of Donor 7 during a period of 3 years. IgG specific for two epitopes on Gad c 1.0101 is induced during the last two years of the study. **(B)** Binding of serum IgG to peptides in the vicinity of the E helix and EF loop of 19 isoallergens/isoforms of parvalbumin. **(C)** Sequences of the peptide corresponding to the E helix and EF loop of parvalbumins, some of which are highly recognized. The lysine (K) found in all peptides bound by IgG of the subject is boxed (cyan). **(D)** Structure of Gal m 1 (PDB: 2MBX). The recognized peptide is highlighted in cyan. The lysine found in all IgG-bound peptides is highlighted (arrow). The numbers of the first residue of peptides are defined in **Figure 1**.

**Figure 9:**
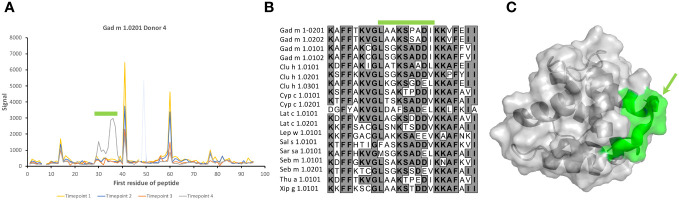
Recognition of peptides representing the BC junction of Gad m 1.0201 induced only in a late sample of one subject. **(A)** Peptide binding profile of Gad m 1.0201 peptides. The numbers of the first residue of peptides are defined in **Figure 1**. Peptides representing the epitope (minimal peptide overlap: LAAKSPADI) of the loop are highlighted with a green bar. The singular high signal in peptide 48 of one sample is considered a technical artifact, as it does not meet the criteria set in section 3.1 to represent a positive signal. **(B)** Sequences of parvalbumins within and in the vicinity of the BC loop. The minimal epitope, a sequence that among these allergens is uniquely found in Gad m 1.0201, is outlined with a green bar. **(C)** The minimal epitope (green) mapped on a structure of Gad m 1 (PDB: 2MBX). The proline that separates the identified epitope sequence from the otherwise identical sequence of Gad m 1.0202 is highlighted by an arrow.

Although reactivity patterns to fish β-parvalbumin peptides tended to be conserved in a given subject during the 3-year study period, specificities occasionally arose or changed substantially during the course of the study. Such novel or greatly increased reactivities were introduced in multiple locations along the β-parvalbumin sequence, as summarized in **Table 2** and outlined in detail in **Figures 2**, **8**, **9**; **Supplementary Figures 1**, **5**. There is thus opportunity for the immune system to evolve the humoral response to fish β-parvalbumins over time.

**Table 2:** Examples of fish β-parvalbumin peptide reactivities induced during the course of the study.

Donor	Allergen	Isotype/subclass	Minimal epitope (colour in structure)	Epitope location	Illustration	
7	Gad c 1.0101	IgG	FDEDGFYAK (blue)	B helix	**Figure 8A**	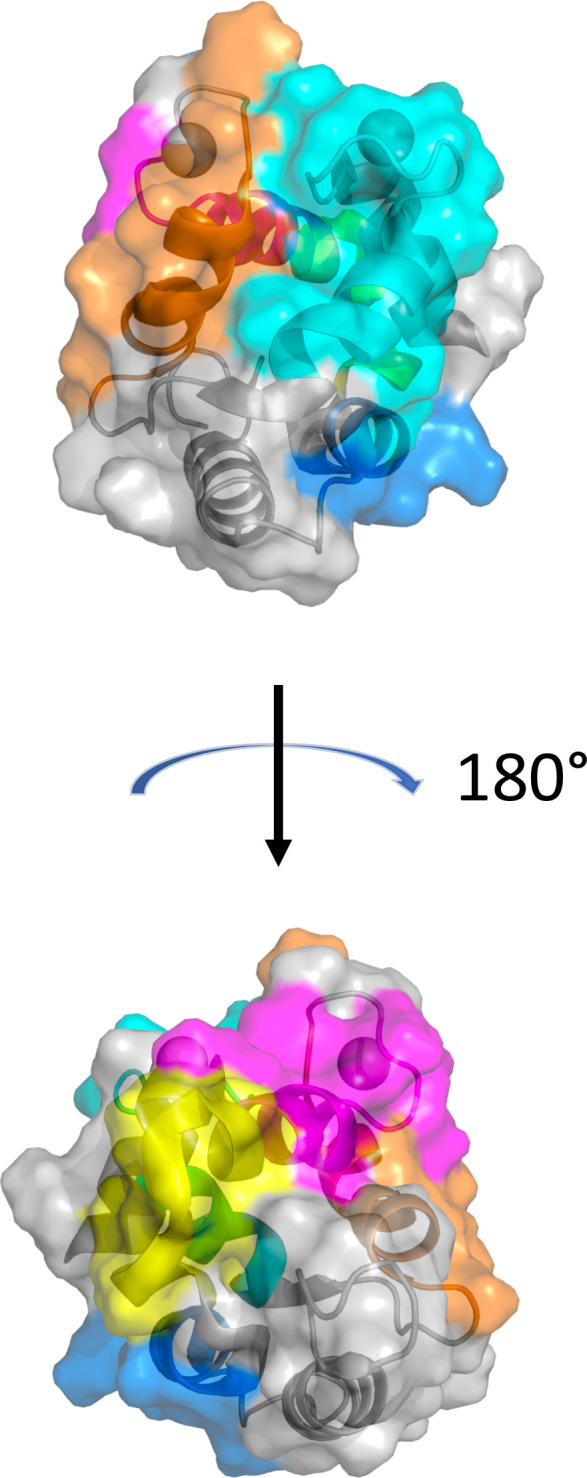
4	Gad m 1.0201	IgG	LAAKSPADI (yellow)	BC loop and C helix N-terminus	**Figure 9**	
6	Thu a 1.0101	IgG	LAAKTPEDI	BC loop and C helix N-terminus	**Supplementary Figure 5B**	
6	Gad c 1.0101	IgG	IADEDK	C helix C-terminus and CD loop	**Supplementary Figure 5B**	
11	Multiple allergens such as Clu h 1.0101	IgG	KKAFEIIDQDK (magenta)	C helix C-terminus and CD loop	**Supplementary Figure 5A**	
2	Multiple allergens such as Thu a 1.0101	IgG4	FIEEDELKLFLQNFSA (orange)	D helix and flanks	**Figure 2**; **Supplementary Figure 1**	
7	Multiple allergens such as Gad m 1.0201	IgG	ETKVFLKAGDSDGDGK (cyan)	E helix and EF loop	**Figures 8B-D**	

Epitopes are modeled onto the structure of Gad m 1 (PDB: 2MBX) and shown in two orientations with loops holding calcium ions (spheres) positioned on top.

### 3.5 Antibody responses that discriminate between allergen isoforms

**Figure 10:**
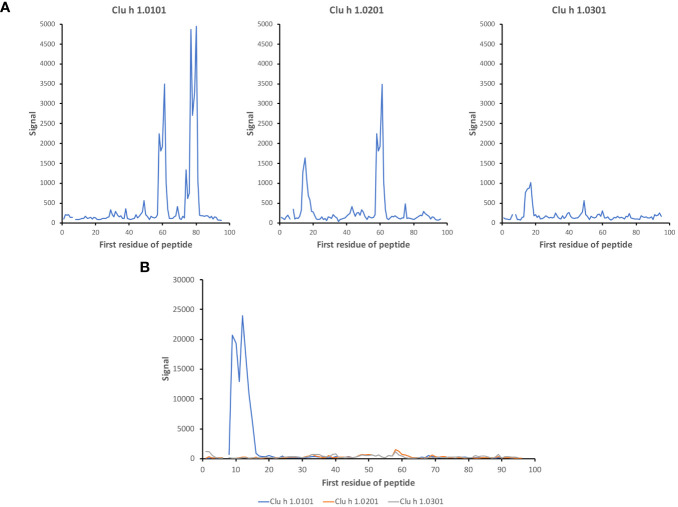
Isoallergen-differentiating epitopes in Clu h 1. Differential recognition of epitopes in isoallergens of Clu h 1 in **(A)** subject 11 and **(B)** subject 12 (time point 3). The numbers of the first residue of peptides are defined in **Figure 1**.

It is conceivable that the composition of allergen isoallergens and isoforms differs between various sources of such products. Accordingly, depending on the composition of specificities in an immune repertoire, contact with different sources of the foods in question may result in different outcomes. Indeed, we identified examples of differential recognition of different isoallergens, for instance in the case of Gad m 1 (**Figures 2**, **5**, **7**; **Supplementary Figure 2**) and Clu h 1 (**Figure 10**). In all, in addition to differences in epitope recognition profiles between different subjects, it is conceivable that differences in the complexity of antibody cross-reactivity against different isoallergens may contribute to different outcomes of allergen-antibody binding in response to various foods.

## 4 Discussion

Complex repertoires of antibodies shape the outcome of their action. Such repertoires and their specificity profiles are in part shaped by the history of antigen exposure. The concept of repertoire imprinting by antigen exposure has been demonstrated for instance in the responses to influenza virus and SARS-CoV-2 (27–29). Other factors, such as an individual’s immunoglobulin germline gene repertoire (30–32), may also shape the repertoire of antibodies that arise upon antigen challenge or vaccination. Arguably, these phenomena may have implications for clinical applications like proper vaccine design (33), including those for use in allergen-specific immunotherapy.

In this study, we demonstrate an individualized pattern of linear epitope recognition related to major fish allergens by IgG. By comparison, the evolution of the epitope recognition profile of a given individual during the three-year study profile was much less extensive. The study cohort that entered into the study were adults. Their anti-β-parvalbumin antibody repertoires were largely established at the time of sampling and little evolution was observed over time, although with some fluctuations. Past studies of immune responses to peptide-defined epitopes of aeroallergens (grass allergens and PR-10-type allergens like Bet v 1) in the same cohort in response to vaccination identified an early establishment of a dominant, individualized epitope reactivity profile that remained relatively stable throughout the final years of the study (20, 21). The epitope recognition of pre-existing aeroallergen-specific humoral immunity similarly remained stable over time. We proposed that allergen-specific B cell clones expanded early during a response and remained dominant over time. Epitopes recognized by aeroallergen-specific IgE were commonly also recognised by the IgG population of the same subject (20, 21), suggesting that there might be a clonal relationship between dominant antibodies over time. Sequencing of antibody-encoding genes, several of which encoded antibodies with defined aeroallergen specificity of these allergic subjects, demonstrated that IgE-encoding allergen-specific clones not uncommonly were maintained during an extended period of time, even throughout the entire 3-year study period (23, 24). Such clones were also identified in the IgG-encoding repertoire, suggesting that a common reservoir of cells might populate plasma cell pools that encode both allergen-specific IgG and IgE. A common clonal origin of multiple peanut allergen-specific IgG and IgE has similarly been observed (34). There is thus potential for studies of the IgG response to also provide information on potential IgE specificities that may develop in case such responses are induced in a given subject. We postulate that the same holds also for clones specific for β-parvalbumins both in terms of IgG and IgE responses, although this remains to be experimentally verified.

A high-content peptide epitope mapping study demonstrated that food allergen-specific IgG responses were shaped by dietary intake (22). The study exploited peptide display on T7 phage to build a reference map of immunogenic epitopes on food allergens and other allergens. All peptide-based epitope mapping technology, either in low- or high-content form, has the drawback of mainly targeting linear, commonly unfolded epitopes. It is, however, reasonable to believe that principles like epitope stability over time, as identified in the present study, are similar for conformational and linear epitopes. The high throughput format exploited herein consists of 16-mer peptides, longer than many other peptide microarrays, which provides opportunities for the identification of some structure-defined epitopes as some peptides will cover entire secondary structure elements of β-parvalbumins, like their α-helices. Furthermore, the analytical format allows for the display of essentially every possible peptide of all allergens included onto the microarray, i.e., with a 15 residue overlap between adjacent 16-mer peptides. A larger offset between adjacent peptides might have prevented the detection of many epitopes that require a longer peptide sequence (see for instance **Figures 2**, **8**, **9**; **Supplementary Figure 1**). The phage-displayed peptide library (22) was able to display longer peptides. However, its resolution in terms of epitope identification is likely lower as each allergen is covered by fewer peptides. The sensitivities of the different assays might also differ. For instance, the commonly detected binding to peptides that carry the C-terminal part of Gad c 1.0101 (**Figure 4**; **Supplementary Figure 4**) was not seen in the samples collected from >1000 individuals investigated by Levitian et al. (22). In contrast, the identification of IgG reactivity to C-terminal peptides of Gad m 1.02 by our peptide microarray agreed with another past study that identified substantial IgE binding to C-terminal peptides of Gad m 1.02 (8). In all, methodological concordance was identified, but further benchmarking of different epitope mapping technologies is warranted to identify best practice for future large-scale epitope mapping of fish parvalbumins.

As outlined above, the immune response to β-parvalbumins is likely shaped by the set of parvalbumins present in the fish species that an individual is exposed to. Salmon (*Salmo salar*), herring (*Clupea harengus*), cod (*Gadus morhua*), skipjack tuna (*Katsuwonus pelamis*/*Euthynnus pelamis*), Alaska pollock (*Theragra calcogramma*), Atlantic mackerel (*Scomber scombrus*), saithe (*Pollachius virens*), and rainbow trout (*Onchorhynchus mykiss*) were the most extensively consumed fish in Sweden, the country of collection of the study’s clinical samples, during 2016 (35). Indeed, the common reactivity to the C-terminus of cod β-parvalbumin suggests the importance of gadiformes in the generation of humoral immunity in the investigated population. As the microarray also carried peptides of a β-parvalbumin of a phylogenetically distinct species (*Rana esculenta* (edible frog)) it was possible to identify common as well as unique epitopes in that species. Consequently, the possibility that β-parvalbumins of species distinct from fish also shape the immune response to fish β-parvalbumins warrants further investigation.

The sequences of the found epitopes also shed some light on how allergens are presented to the immune system. As food components, allergens transit through the gastrointestinal tract, suffer pH- and ligand-binding-induced structural changes, and become exposed to a variety of proteases it is anticipated that fish β-parvalbumins would be presented as a complex mixture of antigens. The core of several of the epitopes herein identified contain F, Y, L, and K residues whose C-terminal peptide bond should have been cleaved during digestion and prevent their immunization capacity. Notwithstanding, some of these epitopes are located in regions known to aggregate (7), and others in segments rich in D and E residues which can bind Ca^2+^ and inhibit the activity of proteases such as trypsin (36).

The sequences of the complete set of parvalbumin allergens from the herein studied and other species to which consumers are exposed are not available. The Genome 10K project (37) represents one effort that will enable the discovery of novel parvalbumins, and others have already been characterized (*e.g.*, https://www.allergome.org), but are not yet incorporated into the WHO/IUIS Allergen Nomenclature Sub-Committee’s Allergen Nomenclature Database. We envisage that complete parvalbumin allergen and peptide resources will allow us to identify dominant parvalbumin immunogens and epitopes. It will be important to follow antibody specificities as they develop also early in life, using such complete sets of allergens and allergen peptides to fully understand these processes. Even in view of the current limitations we are already able to decipher the immune repertoires and to demonstrate the aspects of diversity of responses that define parvalbumin humoral immunity in a population.

## Data availability statement

The data analyzed in this study is subject to the following licenses/restrictions: Data is available directly from the authors upon reasonable request. Requests to access these datasets should be directed to mats.ohlin@immun.lth.se.

## Ethics statement

The studies involving humans were approved by Regional Ethical Board at Lund University. The studies were conducted in accordance with the local legislation and institutional requirements. The participants provided their written informed consent to participate in this study.

## Author contributions

EF: Data curation, Formal analysis, Investigation, Methodology, Software, Validation, Visualization, Writing – review & editing. LT: Conceptualization, Data curation, Formal analysis, Investigation, Methodology, Software, Supervision, Validation, Writing – review & editing. LG: Formal analysis, Investigation, Resources, Supervision, Writing – review & editing. MG: Conceptualization, Investigation, Methodology, Validation, Visualization, Writing – review & editing. MO: Conceptualization, Data curation, Formal analysis, Funding acquisition, Methodology, Resources, Supervision, Validation, Visualization, Writing – original draft, Writing – review & editing.

## References

[1] Hefle SL Nordlee JA Taylor SL. Allergenic foods. Crit Rev Food Sci Nutr. (1996) 36 Suppl:S69–89. doi: 10.1080/10408399609527760 8959379

[2] Chang F Eng L Chang C. Food allergy labeling laws: International guidelines for residents and travelers. Clin Rev Allergy Immunol. (2023) 65:148–65. doi: 10.1007/s12016-023-08960-6 PMC10169132 37160543

[3] Elsayed S Aas K. Characterization of a major allergen (cod). Observations on effect of denaturation on the allergenic activity. J Allergy. (1971) 47:283–91. doi: 10.1016/S0091-6749(71)80006-4 5280452

[4] Dramburg S Hilger C Santos AF de Las Vecillas L Aalberse RC Acevedo N. EAACI molecular allergology user's guide 2.0. Pediatr Allergy Immunol. (2023) 34 Suppl 28:e13854. doi: 10.1111/pai.13854 37186333

[5] Kretsinger RH. Structure and evolution of calcium-modulated proteins. CRC Crit Rev Biochem. (1980) 8:119–74. doi: 10.3109/10409238009105467 6105043

[6] Kolaskar AS Tongaonkar PC. A semi-empirical method for prediction of antigenic determinants on protein antigens. FEBS Lett. (1990) 276:172–4. doi: 10.1016/0014-5793(90)80535-Q 1702393

[7] Sanchez R Martinez J Castro A Pedrosa M Quirce S Rodriguez-Perez R. The amyloid fold of Gad m 1 epitopes governs IgE binding. Sci Rep. (2016) 6:32801. doi: 10.1038/srep32801 27597317 PMC5011719

[8] Perez-Gordo M Pastor-Vargas C Lin J Bardina L Cases B Ibanez MD. Epitope mapping of the major allergen from Atlantic cod in Spanish population reveals different IgE-binding patterns. Mol Nutr Food Res. (2013) 57:1283–90. doi: 10.1002/mnfr.201200332 23554100

[9] Hjort C Schiøtz PO Ohlin M Wurtzen PA Christensen LH Hoffmann HJ. The number and affinity of productive IgE pairs determine allergen activation of mast cells. J Allergy Clin Immunol. (2017) 140:1167–70.e2. doi: 10.1016/j.jaci.2017.04.014 28479333

[10] Christensen LH Holm J Lund G Riise E Lund K. Several distinct properties of the IgE repertoire determine effector cell degranulation in response to allergen challenge. J Allergy Clin Immunol. (2008) 122:298–304. doi: 10.1016/j.jaci.2008.05.026 18572230

[11] Gadermaier E James LK Shamji MH Blatt K Fauland K Zieglmayer P. Epitope specificity determines cross-protection of a SIT-induced IgG4 antibody. Allergy. (2016) 71:36–46. doi: 10.1111/all.12710 26221749 PMC4716291

[12] Shamji MH Valenta R Jardetzky T Verhasselt V Durham SR Wurtzen PA. The role of allergen-specific IgE, IgG and IgA in allergic disease. Allergy. (2021) 76:3627–41. doi: 10.1111/all.14908 PMC8601105 33999439

[13] Santos AF James LK Bahnson HT Shamji MH Couto-Francisco NC Islam S. IgG4 inhibits peanut-induced basophil and mast cell activation in peanut-tolerant children sensitized to peanut major allergens. J Allergy Clin Immunol. (2015) 135:1249–56. doi: 10.1016/j.jaci.2015.01.012 PMC4418748 25670011

[14] Valenta R Karaulov A Niederberger V Zhernov Y Elisyutina O Campana R. Allergen extracts for *in vivo* diagnosis and treatment of allergy: Is there a future? J Allergy Clin Immunol Pract. (2018) 6:1845–55.e2. doi: 10.1016/j.jaip.2018.08.032 30297269 PMC6390933

[15] Jiang X Rao Q. Effect of processing on fish protein antigenicity and allergenicity. Foods. (2021) 10:969. doi: 10.3390/foods10050969 33925068 PMC8145695

[16] Gieras A Linhart B Roux KH Dutta M Khodoun M Zafred D. IgE epitope proximity determines immune complex shape and effector cell activation capacity. J Allergy Clin Immunol. (2016) 137:1557–65. doi: 10.1016/j.jaci.2015.08.055 PMC4890651 26684291

[17] Pomes A Davies JM Gadermaier G Hilger C Holzhauser T Lidholm J. WHO/IUIS Allergen Nomenclature: Providing a common language. Mol Immunol. (2018) 100:3–13. doi: 10.1016/j.molimm.2018.03.003 29625844 PMC6019191

[18] Cia G Pucci F Rooman M. Critical review of conformational B-cell epitope prediction methods. Brief Bioinform. (2023) 24:bbac567. doi: 10.1093/bib/bbac567 36611255

[19] Perez-Gordo M Lin J Bardina L Pastor-Vargas C Cases B Vivanco F. Epitope mapping of Atlantic salmon major allergen by peptide microarray immunoassay. Int Arch Allergy Immunol. (2012) 157:31–40. doi: 10.1159/000324677 21894026

[20] Mikus M Zandian A Sjöberg R Hamsten C Forsström B Andersson M. Allergome-wide peptide microarrays enable epitope deconvolution in allergen-specific immunotherapy. J Allergy Clin Immunol. (2021) 147:1077–86. doi: 10.1016/j.jaci.2020.08.002 32791163

[21] Thörnqvist L Sjöberg R Greiff L van Hage M Ohlin M. Linear epitope binding patterns of grass pollen-specific antibodies in allergy and in response to allergen-specific immunotherapy. Front Allergy. (2022) 3:859126. doi: 10.3389/falgy.2022.859126 35769580 PMC9234942

[22] Leviatan S Vogl T Klompus S Kalka IN Weinberger A Segal E. Allergenic food protein consumption is associated with systemic IgG antibody responses in non-allergic individuals. Immunity. (2022) 55:2454–69.e6. doi: 10.1016/j.immuni.2022.11.004 36473469 PMC12103637

[23] Levin M King JJ Glanville J Jackson KJ Looney TJ Hoh RA. Persistence and evolution of allergen-specific IgE repertoires during subcutaneous specific immunotherapy. J Allergy Clin Immunol. (2016) 137:1535–44. doi: 10.1016/j.jaci.2015.09.027 PMC5010428 26559321

[24] Hoh RA Thörnqvist L Yang F Godzwon M King JJ Lee JY. Clonal evolution and stereotyped sequences of human IgE lineages in aeroallergen-specific immunotherapy. J Allergy Clin Immunol. (2023) 152:214–29. doi: 10.1016/j.jaci.2023.02.009 PMC12898903 36828082

[25] Dhanda SK Mahajan S Paul S Yan Z Kim H Jespersen MC. IEDB-AR: immune epitope database-analysis resource in 2019. Nucleic Acids Res. (2019) 47:W502–W6. doi: 10.1093/nar/gkz452 PMC6602498 31114900

[26] Willforss J Siino V Levander F. OmicLoupe: facilitating biological discovery by interactive exploration of multiple omic datasets and statistical comparisons. BMC Bioinf. (2021) 22:107. doi: 10.1186/s12859-021-04043-5 PMC7931979 33663372

[27] Gostic KM Ambrose M Worobey M Lloyd-Smith JO. Potent protection against H5N1 and H7N9 influenza via childhood hemagglutinin imprinting. Science. (2016) 354:722–6. doi: 10.1126/science.aag1322 PMC5134739 27846599

[28] Röltgen K Nielsen SCA Silva O Younes SF Zaslavsky M Costales C. Immune imprinting, breadth of variant recognition, and germinal center response in human SARS-CoV-2 infection and vaccination. Cell. (2022) 185:1025–40.e14. doi: 10.1016/j.cell.2022.01.018 35148837 PMC8786601

[29] Tas JMJ Koo JH Lin YC Xie Z Steichen JM Jackson AM. Antibodies from primary humoral responses modulate the recruitment of naive B cells during secondary responses. Immunity. (2022) 55:1856–71.e6. doi: 10.1016/j.immuni.2022.07.020 35987201 PMC9350677

[30] Sangesland M Torrents de la Pena A Boyoglu-Barnum S Ronsard L Mohamed FAN Moreno TB. Allelic polymorphism controls autoreactivity and vaccine elicitation of human broadly neutralizing antibodies against influenza virus. Immunity. (2022) 55:1693–709.e8. doi: 10.1016/j.immuni.2022.07.006 35952670 PMC9474600

[31] Avnir Y Watson CT Glanville J Peterson EC Tallarico AS Bennett AS. IGHV1-69 polymorphism modulates anti-influenza antibody repertoires, correlates with IGHV utilization shifts and varies by ethnicity. Sci Rep. (2016) 6:20842. doi: 10.1038/srep20842 26880249 PMC4754645

[32] Lee JH Toy L Kos JT Safonova Y Schief WR Havenar-Daughton C. Vaccine genetics of IGHV1-2 VRC01-class broadly neutralizing antibody precursor naive human B cells. NPJ Vaccines. (2021) 6:113. doi: 10.1038/s41541-021-00376-7 34489473 PMC8421370

[33] Wheatley AK Fox A Tan HX Juno JA Davenport MP Subbarao K. Immune imprinting and SARS-CoV-2 vaccine design. Trends Immunol. (2021) 42:956–9. doi: 10.1016/j.it.2021.09.001 PMC8440232 34580004

[34] Hoh RA Joshi SA Liu Y Wang C Roskin KM Lee JY. Single B-cell deconvolution of peanut-specific antibody responses in allergic patients. J Allergy Clin Immunol. (2016) 137:157–67. doi: 10.1016/j.jaci.2015.05.029 PMC4699867 26152318

[35] Borthwick L Bergman K Ziegler F. Svensk konsumtion av sjömat. Göteborg: RISE Research Institutes of Sweden AB (2019).

[36] Sipos T Merkel JR. An effect of calcium ions on the activity, heat stability, and structure of trypsin. Biochemistry. (1970) 9:2766–75. doi: 10.1021/bi00816a003 5466615

[37] Rhie A McCarthy SA Fedrigo O Damas J Formenti G Koren S. Towards complete and error-free genome assemblies of all vertebrate species. Nature. (2021) 592:737–46. doi: 10.1038/s41586-021-03451-0 PMC8081667 33911273

