# Supplementary material for: Linear epitopes of bony fish β-parvalbumins
Source: Front Immunol. 2024 Mar 5;15:1293793. doi: 10.3389/fimmu.2024.1293793 (PMC10948427; doi:10.3389/fimmu.2024.1293793)

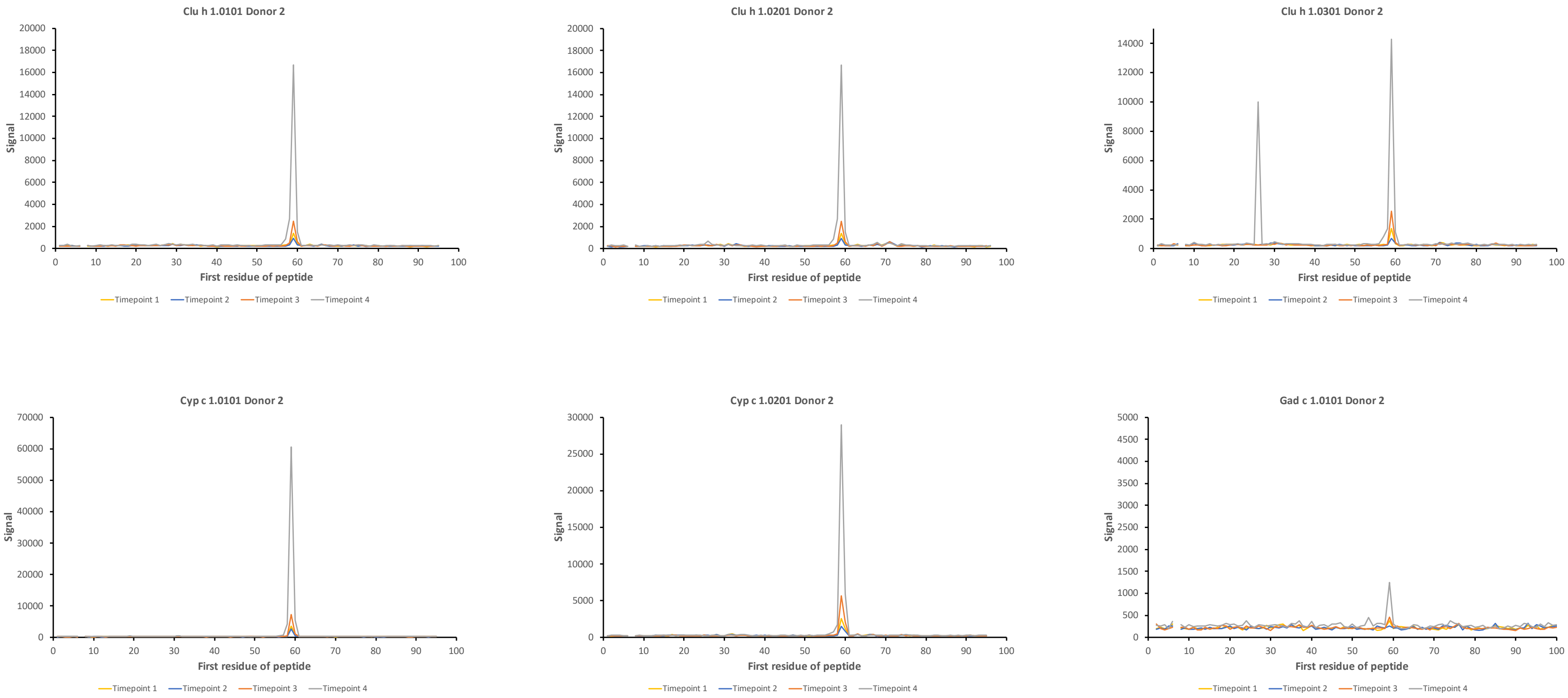

Supplementary Figure 1

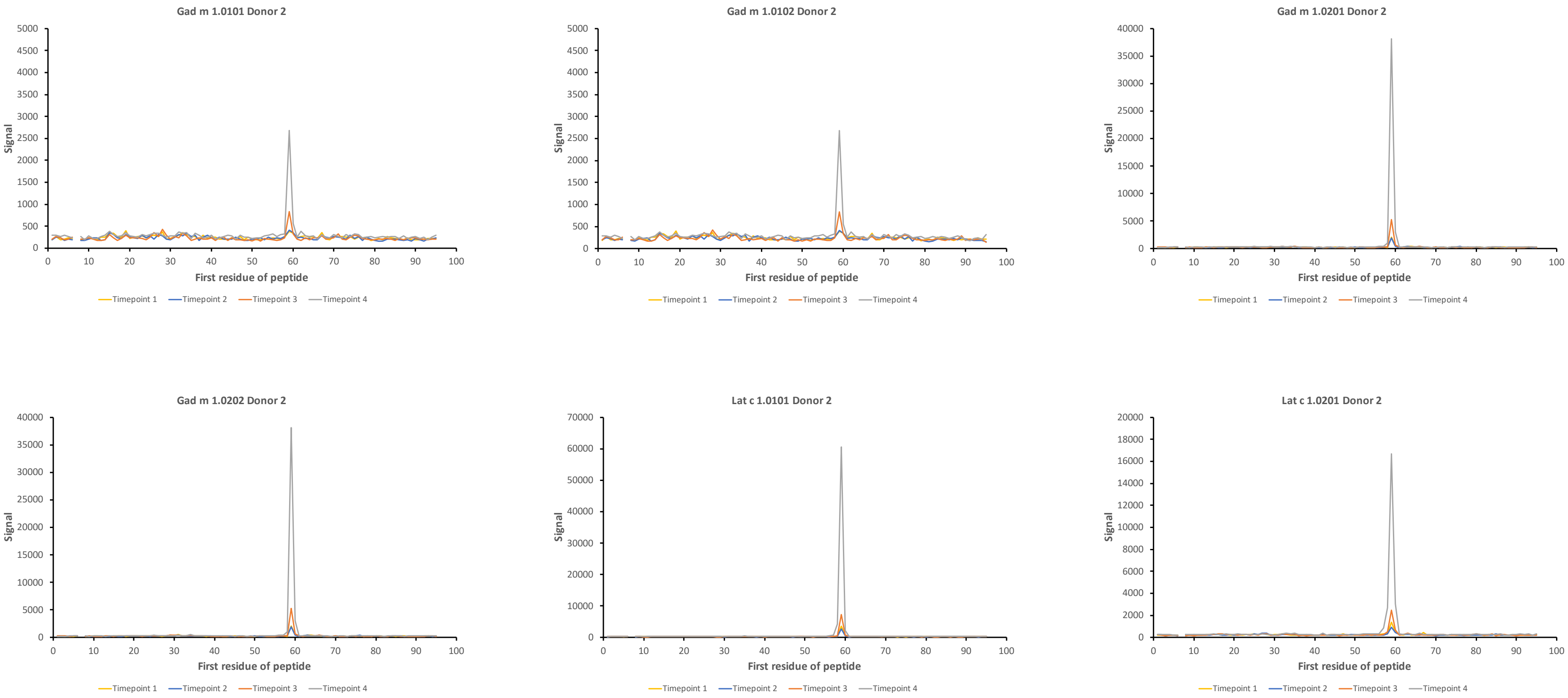

Supplementary Figure 1 (continued)

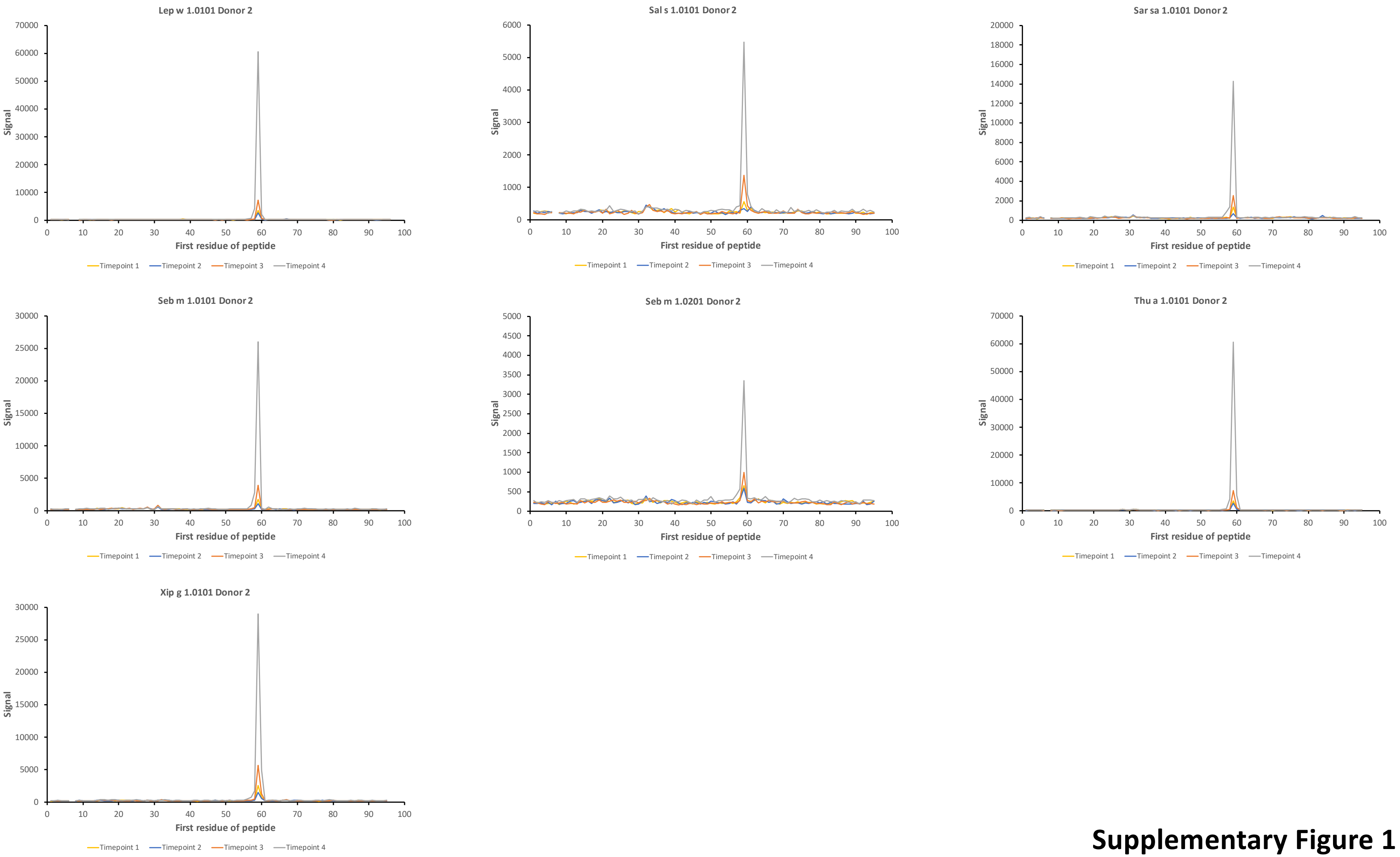

Supplementary Figure 1 (continued)

Supplementary Figure 2

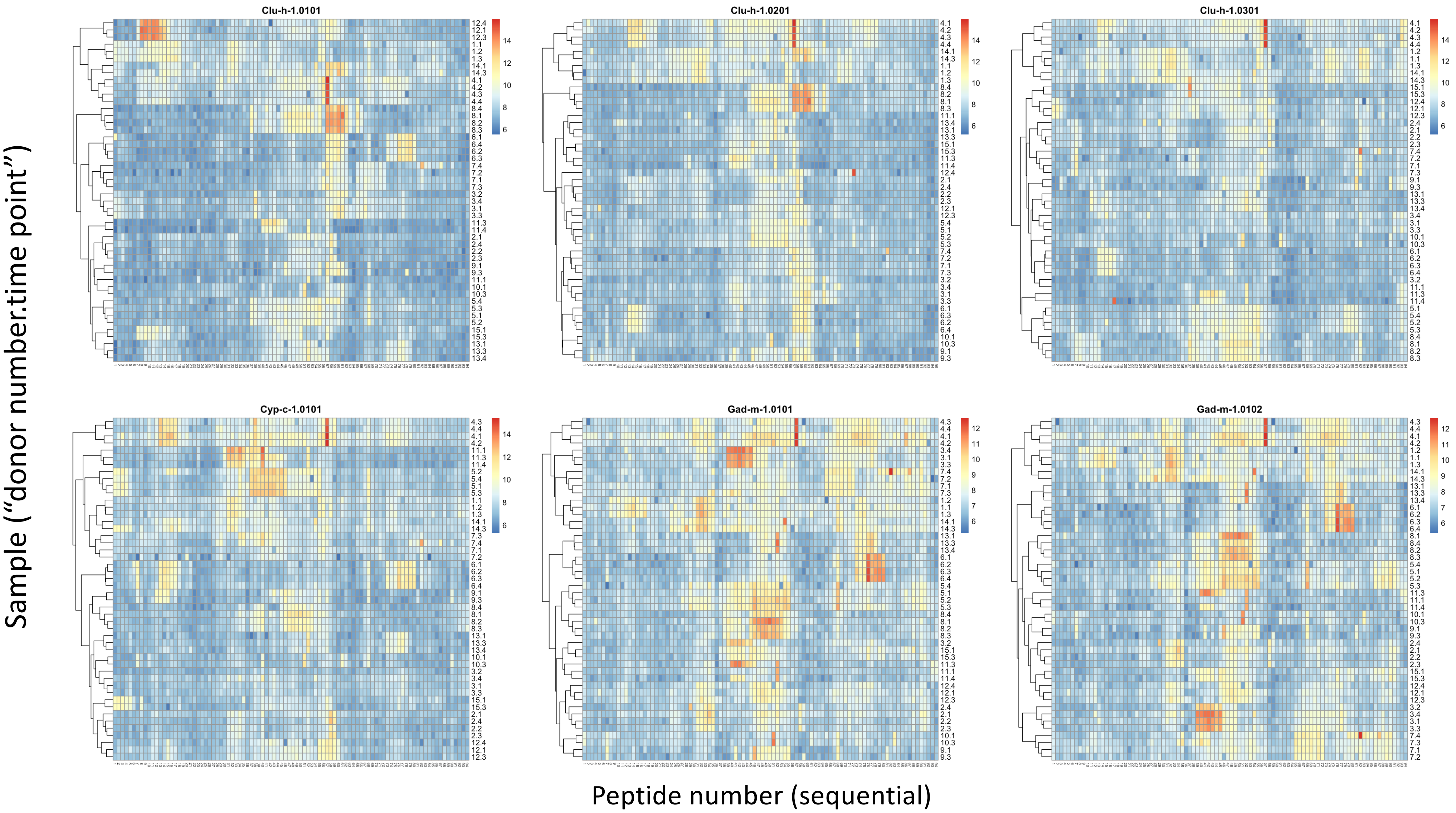

Supplementary Figure 2 (continued)

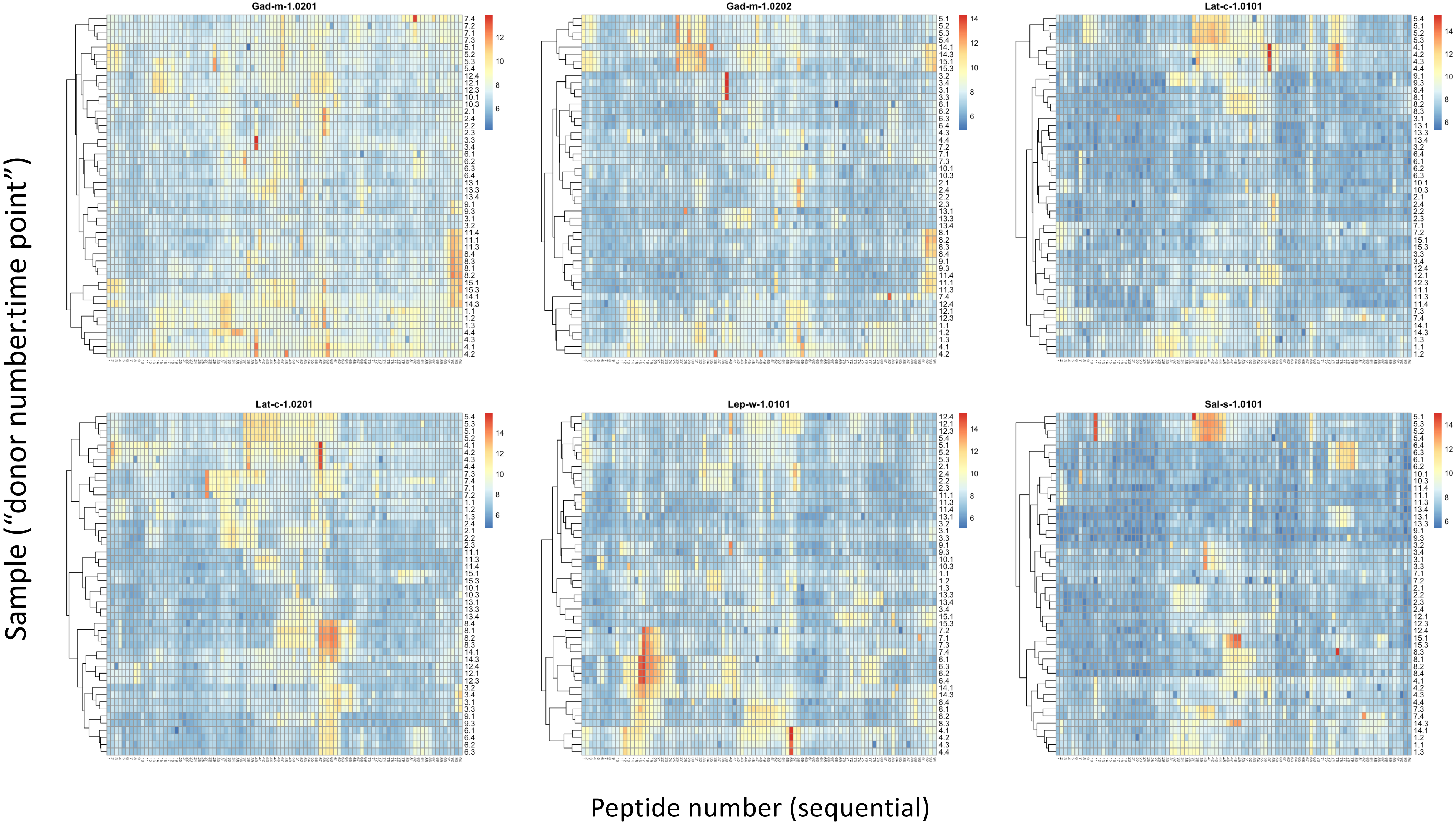

Supplementary Figure 2 (continued)

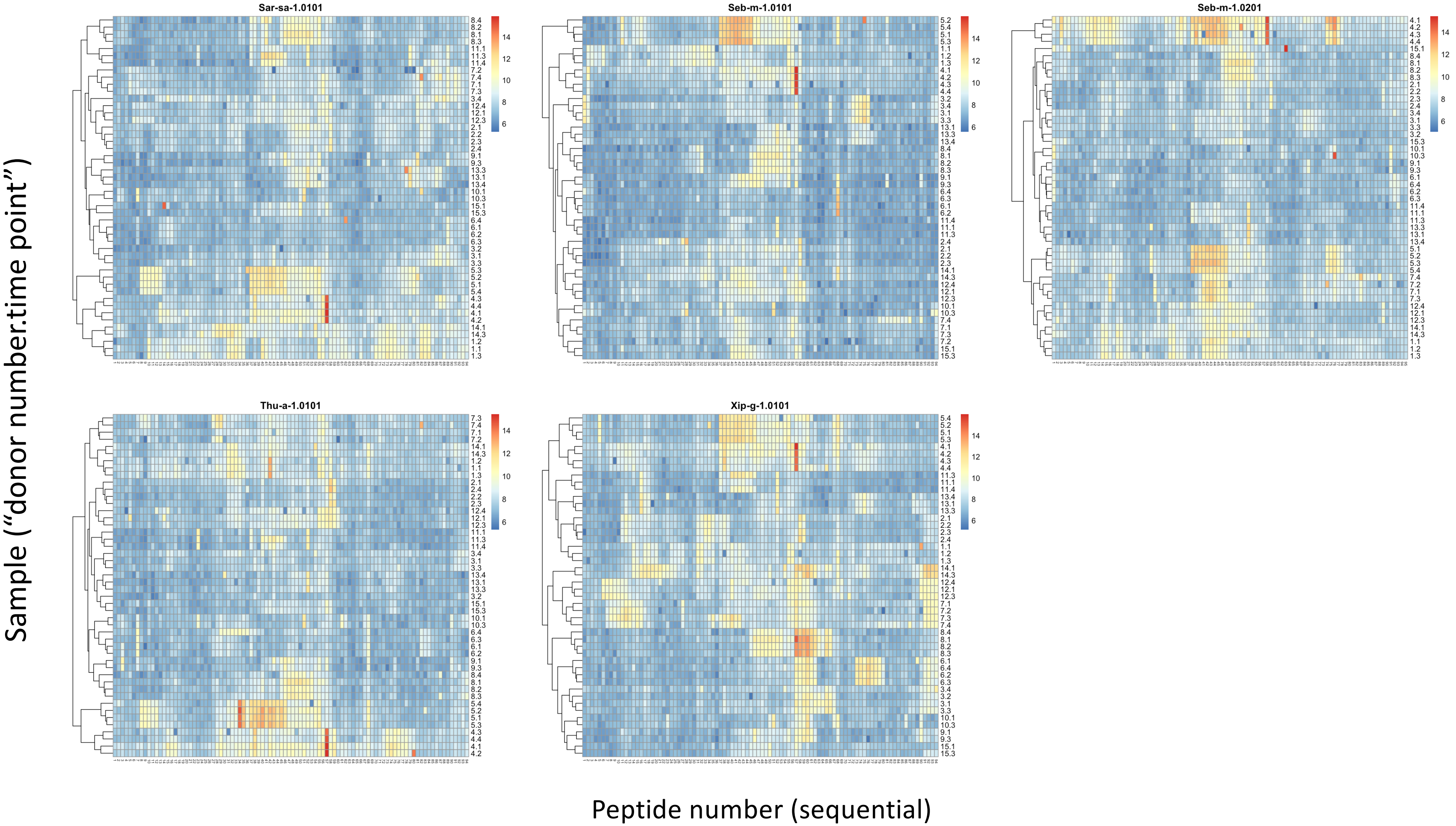

Supplementary Figure 3

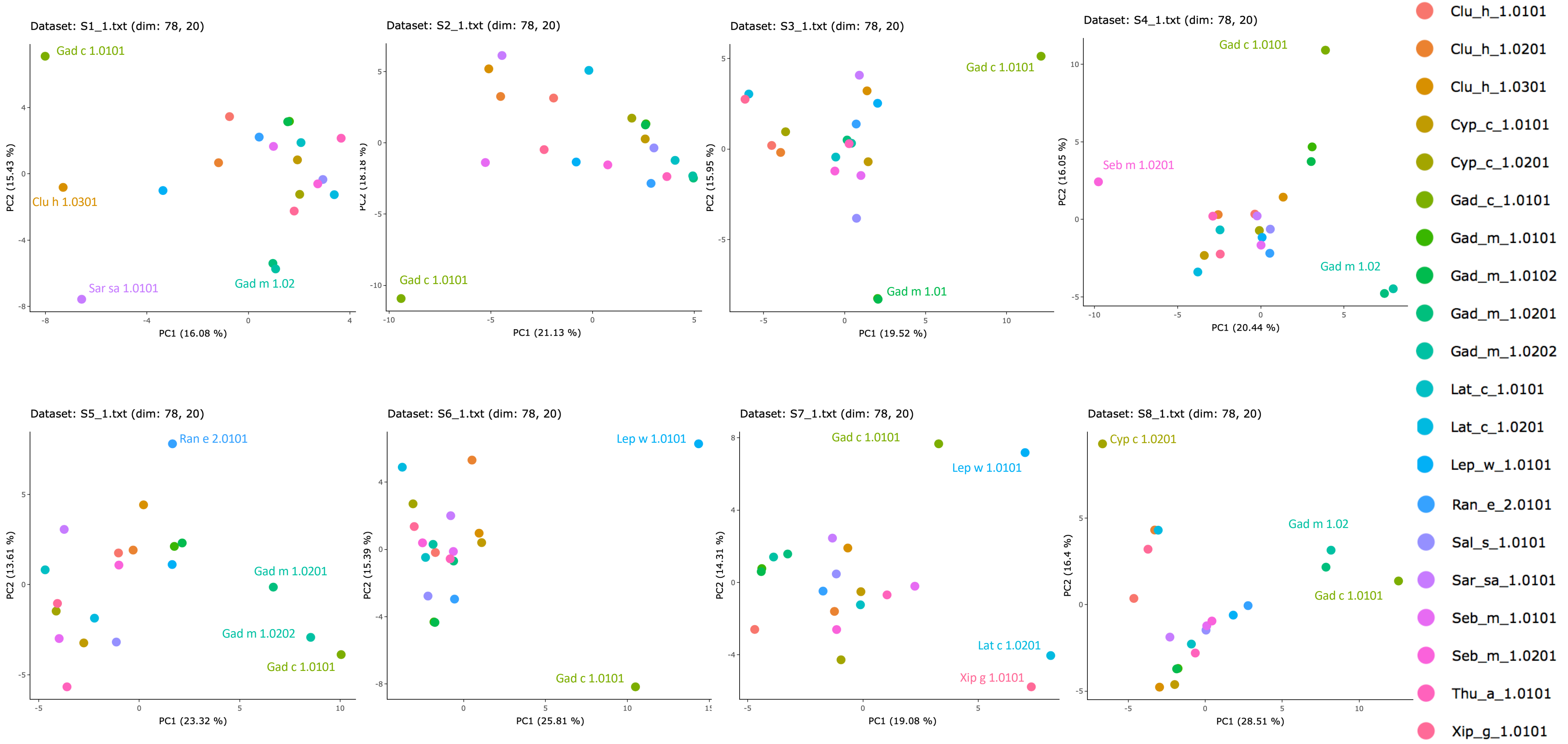

Supplementary Figure 3 (continued)

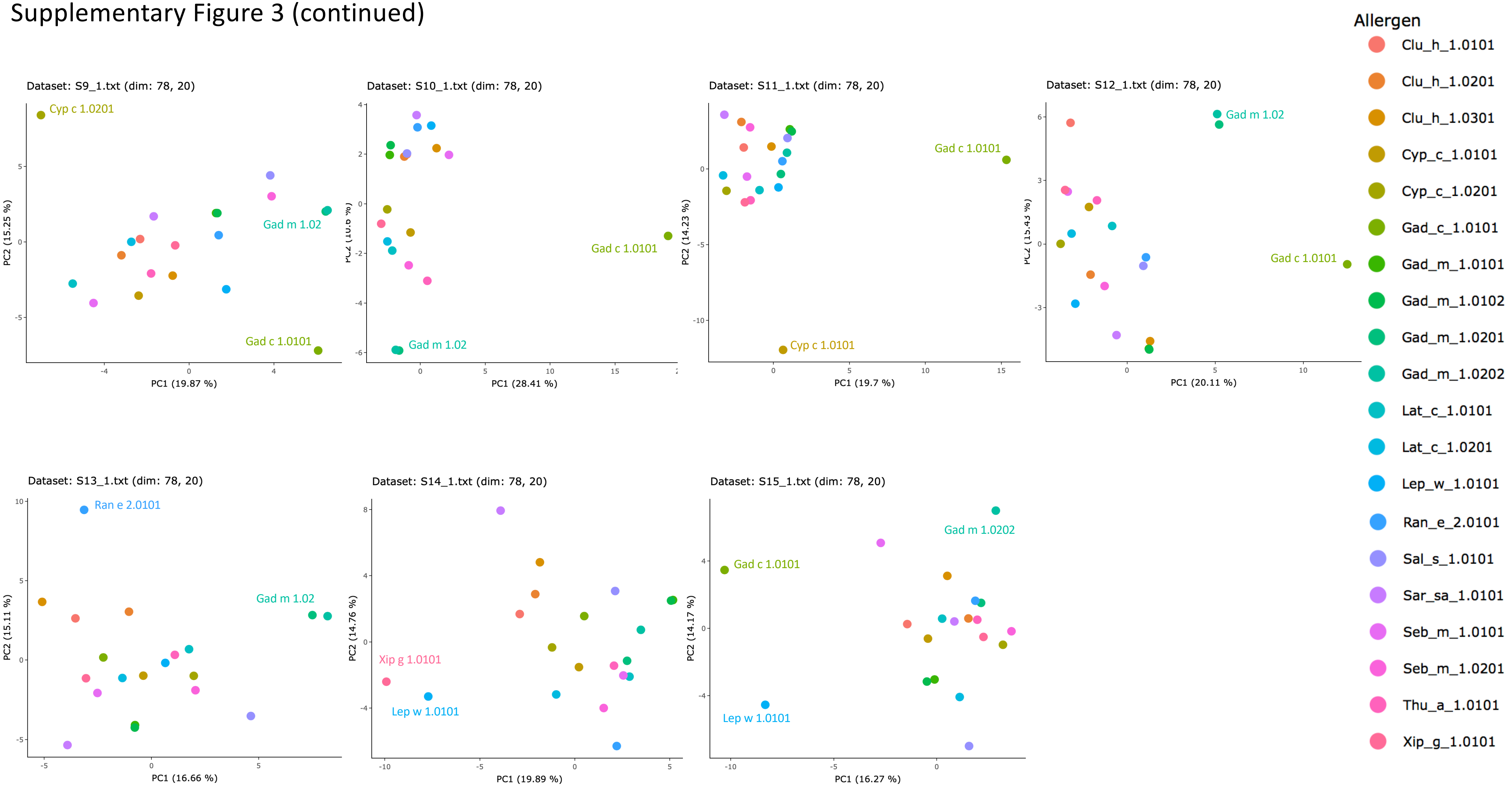

Supplementary Figure 4

A

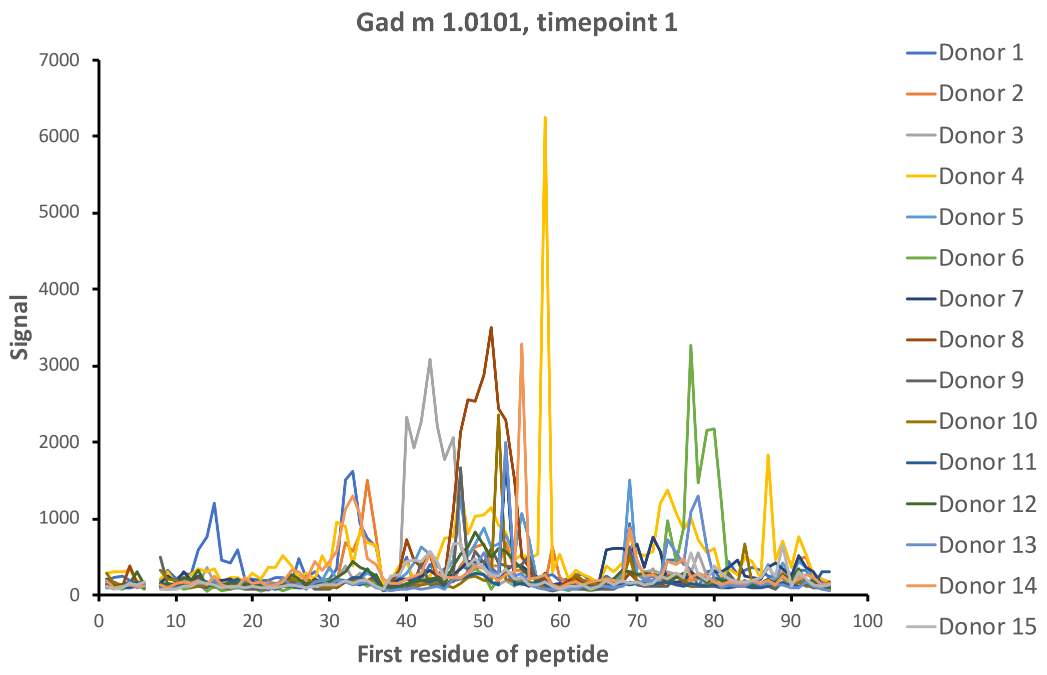

B

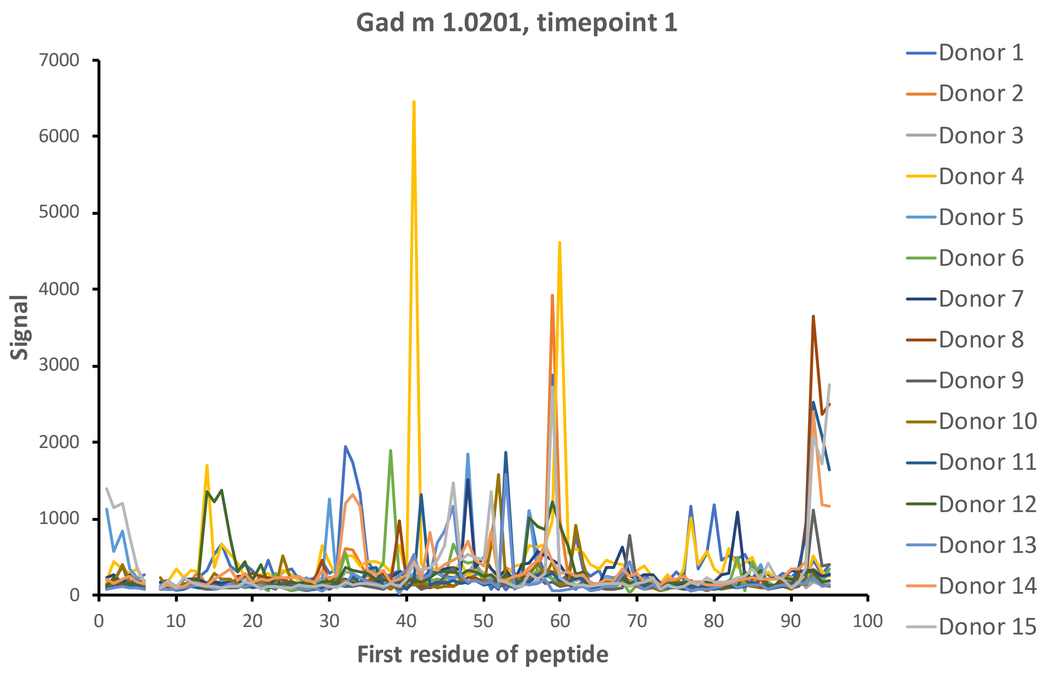

Supplementary Figure 5A

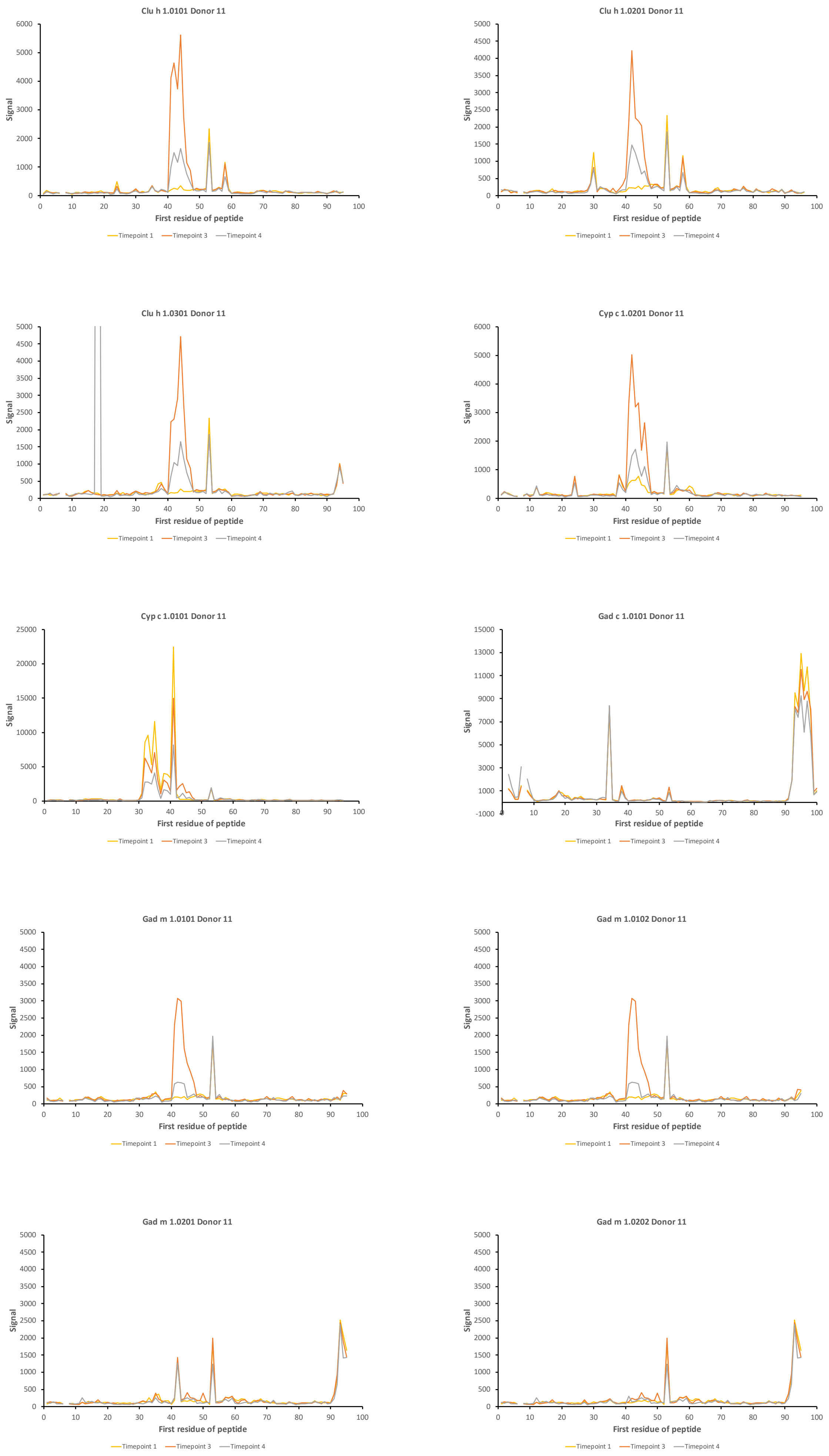

Supplementary Figure 5A (continued)

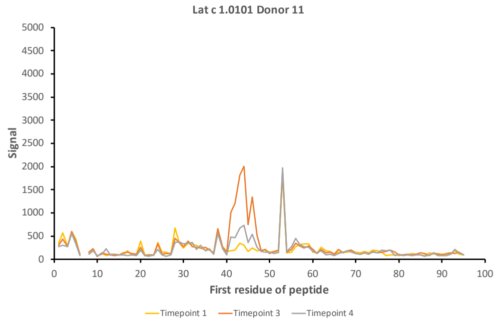

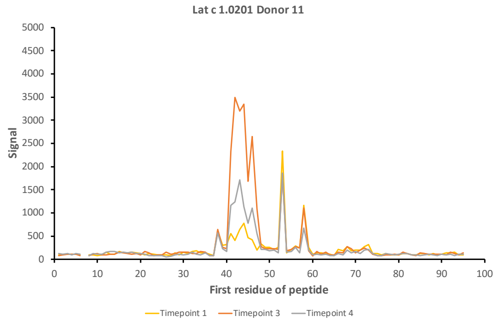

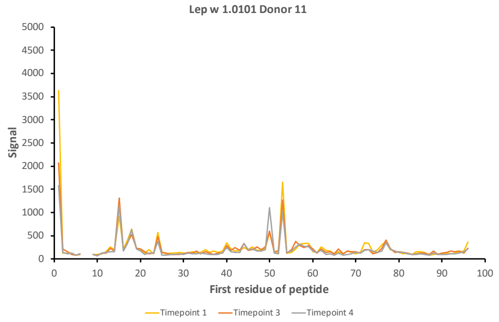

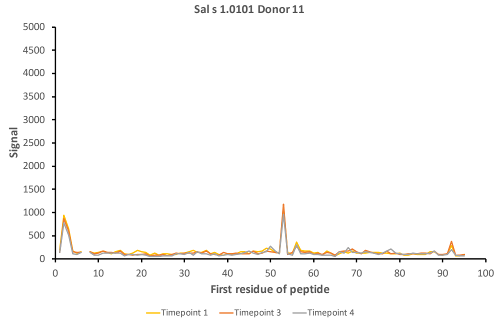

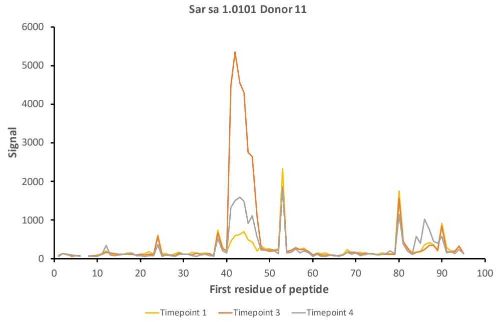

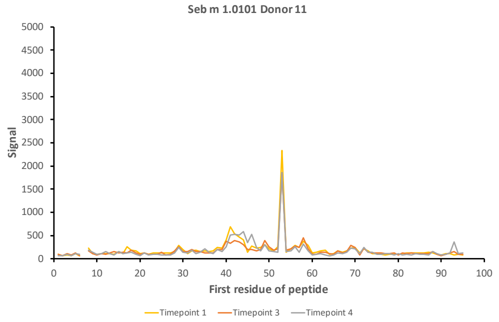

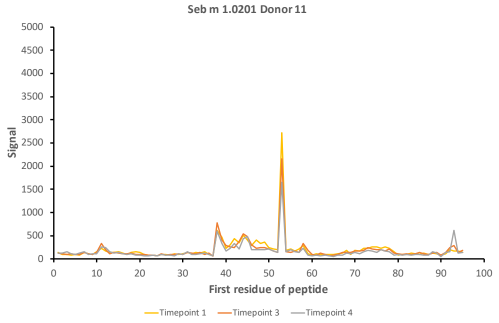

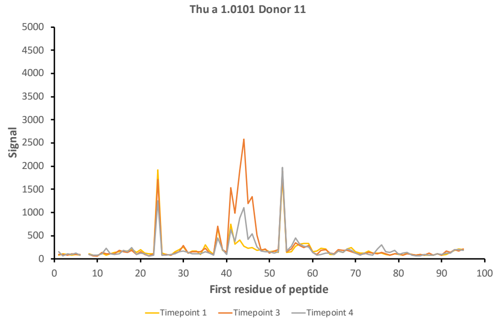

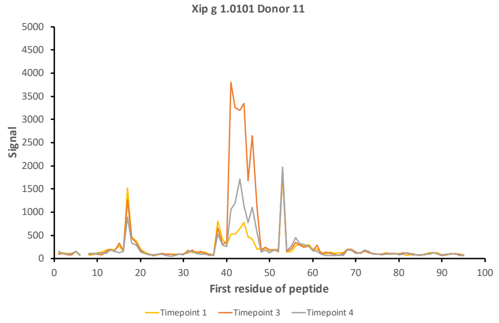

Supplementary Figure 5B

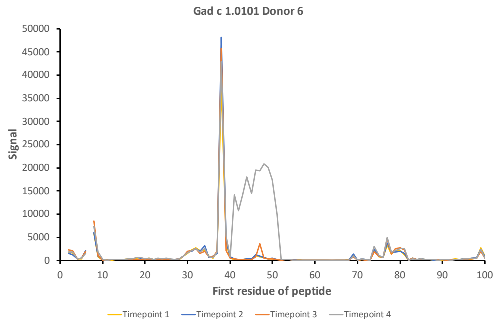

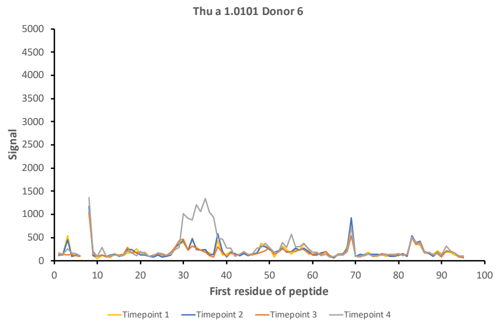

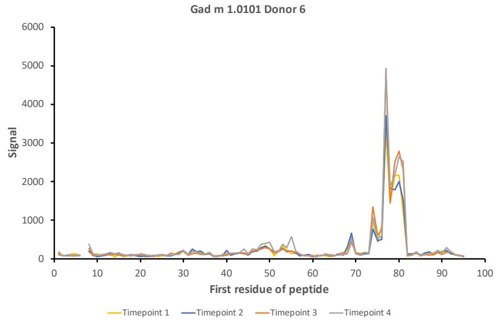

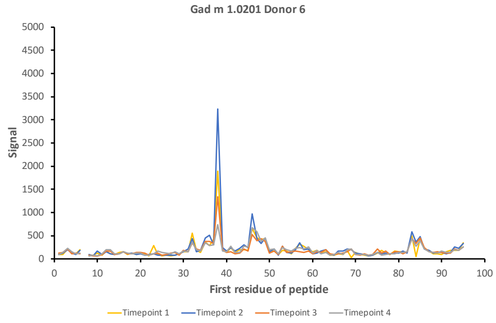

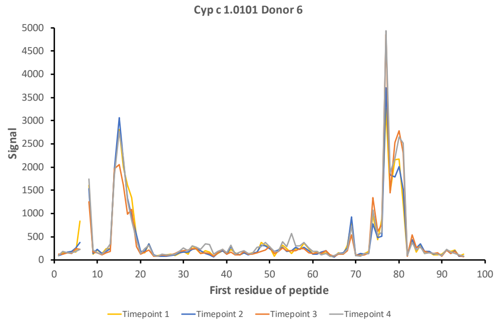

Supplement: Supplementary file 1 [file DataSheet_1.pdf]
